# Development and validation of a recombinant Omp19+31-based indirect hemagglutination assay for serological diagnosis of bovine and ovine brucellosis

**DOI:** 10.14202/vetworld.2026.2512-2530

**Published:** 2026-06-20

**Authors:** Aitbay Bulashev, Alfiya Syzdykova, Anara Kukayeva, Aibek Zhumalin, Guldarigash Kaukabaeva, Mikail Mikailov, Maxat Berdikulov, Bakytkali Ingirbay

**Affiliations:** 1 Department of Animal Husbandry and Biological Sciences, Institute of Animal Science and Veterinary Medicine, Saken Seifullin Kazakh Agrotechnical University, Astana, Kazakhstan; 2 Department of Veterinary Science, Shakarim University of Semey, Semey, Kazakhstan; 3 Caspian Zonal Research Institute of Veterinary Medicine, Federal Agrarian Scientific Center of the Republic of Dagestan, Makhachkala, Russian Federation; 4 Infectious Disease Diagnostic Laboratory, National Reference Center for Veterinary Medicine, Astana, Kazakhstan; 5 Laboratory of Genetic Engineering, National Center for Biotechnology, Astana, Kazakhstan

**Keywords:** animal brucellosis, bovine brucellosis, chimeric recombinant antigen, indirect hemagglutination assay, Omp19, Omp31, ovine brucellosis, serological diagnosis

## Abstract

**Background and Aim:**

Brucellosis remains a major zoonotic disease affecting livestock production and public health worldwide, particularly in endemic regions such as Kazakhstan. Conventional serological assays used for animal brucellosis diagnosis are often limited by cross-reactivity associated with lipopolysaccharide-based antigens, leading to false-positive results and unnecessary culling of animals. The indirect hemagglutination assay (IHA) is a simple and economical serological method; however, its diagnostic specificity requires improvement. This study aimed to develop and validate a recombinant protein-based IHA using sheep erythrocytes sensitized with recombinant Brucella outer membrane and periplasmic proteins for the serological diagnosis of bovine and ovine brucellosis.

**Materials and Methods:**

Recombinant *Brucella* proteins, including outer membrane protein (Omp)19, Omp25, Omp31, their chimeric combinations (Omp19+25, Omp19+31, and Omp25+31), and the periplasmic proteins Bp26 and Cu/ZnSOD, were produced in *Escherichia coli* BL21(DE3). Sheep erythrocytes were sensitized with recombinant proteins to prepare erythrocyte diagnostic reagents for IHA. A total of 1,440 serum samples from cattle and sheep, including samples from brucellosis-affected and brucellosis-free herds, were evaluated using IHA, indirect enzyme-linked immunosorbent assay (ELISA), and Rose Bengal plate test. In addition, biological samples from 32 seropositive cattle subjected to sanitary slaughter were examined by culture isolation and polymerase chain reaction. Diagnostic sensitivity, specificity, and accuracy were calculated with 95% confidence intervals (CI).

**Results:**

Among all tested antigens, the Omp19+31 chimeric protein demonstrated the highest diagnostic performance in IHA. The Omp19+31-based IHA achieved a sensitivity of 92.6% (95% CI: 85.5–96.4), specificity of 100% (95% CI: 99.2–100.0), and overall diagnostic accuracy of 98.8% (95% CI: 97.5–99.5). In the subset of 32 seropositive cattle, the assay detected *Brucella*-specific antibodies in all sera, including in culture-positive cases that were missed by commercial ELISA. The assay predominantly produced strong agglutination reactions and showed superior specificity compared with single-antigen formats. Cross-reactivity analysis confirmed that Omp19 and Omp31 exhibited high specificity toward *Brucella* spp. with minimal reactivity against related Gram-negative bacteria.

**Conclusion:**

The recombinant Omp19+31-based IHA demonstrated excellent specificity, high diagnostic accuracy, and operational simplicity for the serological diagnosis of bovine and ovine brucellosis. The assay represents a promising complementary diagnostic tool for endemic and resource-limited settings where conventional lipopolysaccharide-based assays are limited by cross-reactivity. Further multi-center validation and assessment of differentiating infected from vaccinated animals potential are warranted before broader implementation.

## INTRODUCTION

Brucellosis is a globally distributed zoonosis reported on all inhabited continents, with human cases documented in at least 170 countries, reflecting the ecological adaptability of *Brucella* spp. [1]. The Central Asian republics are among the regions with the highest reported incidence of brucellosis. In Kazakhstan, brucellosis-positive animals have been detected in 56.4% of surveyed rural districts, indicating widespread distribution across the country. Among the examined populations, the prevalence of infection was estimated at 0.7% in cattle and 0.1% in small ruminants [2]. Kazakhstan ranks second in Central Asia in terms of reported brucellosis incidence per 1 million population (116 cases), following Kyrgyzstan (362 cases). Nevertheless, these official statistics are widely considered to underestimate the true burden of the disease [3]. Early diagnosis of animal brucellosis is a cornerstone of integrated veterinary and sanitary control programs aimed at the containment and eventual eradication of the disease. Conventional serological assays, including commercial enzyme-linked immunosorbent assay (ELISA) kits, predominantly target antibodies directed against the smooth lipopolysaccharide (S-LPS) of the *Brucella* cell envelope. However, the high structural similarity of S-LPS epitopes to those of phylogenetically related Gram-negative bacteria leads to antibody cross-reactivity, thereby increasing the risk of false-positive results [4].

Kazakhstan had an unfavorable experience with the implementation of ELISA in the serodiagnosis of animal brucellosis during 2008–2012, when the “test and slaughter” strategy was adopted as the primary control measure. During this period, the number of cattle identified as brucellosis-positive increased several-fold compared with previous years and remained persistently high throughout the ELISA application phase, with no measurable improvement in the epizootic situation [5]. This approach resulted in the potential culling of uninfected animals, primarily due to the high sensitivity but low specificity of ELISA. These shortcomings ultimately led to the discontinuation of ELISA-based screening and a return to classical serological methods for the diagnosis of brucellosis. Given Kazakhstan’s vast territory, ranking ninth worldwide by land area, and the limited technical capacity of many diagnostic laboratories, there is a clear need for a simple, robust, and highly specific diagnostic assay suitable for large-scale screening of animal populations. One such approach may be based on the indirect hemagglutination assay (IHA) employing *Brucella*-specific antigens. While recombinant *Brucella* periplasmic proteins [6–8] and outer membrane proteins (Omps) [9, 10] have been extensively evaluated in ELISA, their use as sensitizing antigens in the IHA remains underexplored. Existing IHA formats have largely relied on LPS or whole-cell antigens, which are inherently prone to cross-reactivity with related Gram-negative bacteria [11].

The present study was designed to address these limitations by developing and evaluating a recombinant protein-based IHA employing sheep erythrocytes sensitized with recombinant *Brucella* Omps and periplasmic proteins, including chimeric antigen constructs. Particular emphasis was placed on assessing the diagnostic utility of the outer membrane protein (Omp)19+31 chimeric protein as a sensitizing antigen for improving assay specificity and diagnostic performance. The study further aimed to compare the performance of the developed IHA with conventional serological methods and i-ELISA using serum samples obtained from cattle and sheep originating from brucellosis-affected and brucellosis-free herds in Kazakhstan.

To our knowledge, this study is among the first systematic efforts to optimize and validate recombinant Omp-based erythrocyte diagnostic reagents for IHA, using both bovine and ovine field sera from a high-burden endemic setting. The proposed approach integrates the operational simplicity and field applicability of IHA with the enhanced specificity of well-defined recombinant protein antigens. The developed IHA is intended as a complementary serological assay to clarify the antibody status of animals with positive or equivocal results obtained by conventional tests. Although brucellosis vaccination is permitted in Kazakhstan, the present study was not designed to assess differentiation between infected and vaccinated animals (DIVA), which remains an important direction for future research.

## MATERIALS AND METHODS

### Ethical approval

All procedures involving animals were conducted in accordance with internationally accepted principles for the care and use of laboratory animals and in compliance with the Interstate Standard GOST 33216-2014 for the ethical treatment of experimental animals. The study protocol was reviewed and approved by the Ethics Committee of the Faculty of Veterinary Science and Animal Husbandry Technology, Saken Seifullin Kazakh Agrotechnical University (KATU), Astana, Kazakhstan (Protocol No. 2, November 3, 2022).

One Kazakh semi-fine-wool ram and two male Soviet Chinchilla rabbits used for erythrocyte collection and antiserum production were maintained under standard husbandry conditions with unrestricted access to feed and water. Animal handling, immunization, blood collection, and monitoring procedures were performed by trained veterinary personnel to minimize stress, discomfort, and pain. Rabbits were monitored daily throughout the immunization period for signs of adverse reactions, and blood samples were collected under appropriate restraint and sedation when required.

The serum samples and biological materials obtained from cattle and sheep were collected as part of routine veterinary surveillance and disease control programs conducted by the Republican Veterinary Laboratory in Kazakhstan. Samples used in this study were analyzed anonymously, and no additional invasive procedures were performed specifically for research purposes. Tissue samples collected from cattle subjected to sanitary slaughter originated from animals removed under official veterinary regulations for brucellosis control and were not slaughtered specifically for this investigation.

All laboratory procedures involving potentially infectious *Brucella* materials, including bacteriological culture and molecular analyses, were performed at the National Reference Center for Veterinary Medicine under biosafety level 3 (BSL-3) containment conditions in accordance with national biosafety regulations. Every effort was made to ensure animal welfare, reduce the number of animals used, and minimize any potential distress throughout the study.

### Study period and location

The study was conducted from March 2023 to October 2025 at the Department of Microbiology and Biotechnology, KATU, Astana, Kazakhstan.

### Study design

This study was designed as a laboratory-based experimental diagnostic validation study aimed at developing and evaluating a recombinant protein-based IHA for the serological diagnosis of bovine and ovine brucellosis. Recombinant *Brucella* proteins, including single and chimeric Omps, were produced and used for erythrocyte sensitization. The diagnostic performance of the developed IHA was evaluated using serum samples obtained from cattle and sheep originating from both brucellosis-affected and brucellosis-free herds/flocks. The assay performance was compared with those of conventional serological tests and i-ELISA. Additional bacteriological and molecular analyses were performed using tissue samples collected from seropositive cattle subjected to sanitary slaughter.

### Microorganism strains

Inactivated strains of *Brucella abortus* 19 and *Brucella melitensis* Rev1, kindly provided by the Research and Production Enterprise (RPE) “Antigen” (Almaty, Kazakhstan), were used as immunogens for the production of the corresponding rabbit antisera.

*Escherichia coli* BL21(DE3) strains obtained in our previous studies were used to produce recombinant Omps, including Omp19 [12], Omp25, and Omp31 [13], as well as chimeric (combined/composite) proteins Omp19+Omp25, Omp19+Omp31, and Omp25+Omp31 [14]. The latter were designed and characterized in our previous work [14] as artificial multi-epitope constructs generated by direct fusion of immunodominant regions of *Brucella* Omps without linker sequences and expressed as single fusion polypeptides. No modifications to the original design were introduced in the present study. *E. coli* strains producing the periplasmic proteins Cu-ZnSOD [15] and Bp26 [16] were kindly provided by Professor S.Z. Eskendirova, National Center for Biotechnology, Astana, Kazakhstan.

### Production of recombinant protein-producing *E. Coli* BL21(DE3) strains

Individual colonies of *E. coli* BL21(DE3) strains expressing recombinant *Brucella* proteins were cultured in Luria–Bertani broth (Sigma-Aldrich Co., Steinheim, Germany) supplemented with kanamycin and/or ampicillin. At the mid-logarithmic phase of growth (optical density at 600 nm, OD_600_ ≈ 0.6), protein expression was induced by the addition of isopropyl β-D-1-thiogalactopyranoside (IPTG; Sigma-Aldrich, St. Louis, MO, USA) to a final concentration of 0.5 mM. The cultures were then incubated at room temperature (23 ± 2°C) for 16 h with shaking. Bacterial cells were harvested by centrifugation at 6,000 × *g* for 7 min at 4°C.

### Isolation and purification of proteins

Recombinant proteins were isolated from the producer strains after ultrasonic cell disruption at cold temperatures. Proteins predominantly expressed as inclusion bodies were solubilized under denaturing conditions and purified by immobilized metal affinity chromatography using a Ni^2+^-charged column, and eluted fractions were monitored spectrophotometrically (ThermoScientific, Waltham, MA, USA). The purity and identity of the recombinant proteins were assessed by sodium dodecyl sulfate–polyacrylamide gel electrophoresis (SDS-PAGE) and further confirmed by western immunoblotting with anti-histidine tag antibodies (Thermo Scientific).

### Determination of antigenicity of recombinant proteins by i-ELISA

The wells of polystyrene microtiter plates (ThermoFisher Scientific, Kandel, Germany) were coated with individual recombinant *Brucella* spp. antigens, including Omps (Omp19, Omp25, and Omp31), their combinations (Omp19+25, Omp19+31, and Omp25+31), as well as the periplasmic proteins Bp26 and Cu-ZnSOD, at a concentration of 1.0 µg/mL in carbonate–bicarbonate buffer (CBB, pH 9.6). Sensitization of the wells with immunogens (*B. abortus* 19 and *B. melitensis* Rev1) was performed using a bacterial cell suspension containing 1 × 10^9^ cells/mL. The plates were incubated overnight at 4°C. Rabbit anti-*Brucella* sera were added to the wells in serial dilutions starting from 1:100 in phosphate-buffered saline (PBS) containing Tween 20 (PBS-T) and incubated at 37°C for 1 h. Goat anti-rabbit IgG (Jackson ImmunoResearch, West Baltimore, MD, USA) was used as the secondary antibody. The endpoint antibody titer was defined as the highest serum dilution at which the OD value exceeded that of the negative rabbit serum diluted 1:100 by at least two-fold.

### Standard sera

*Brucella*-positive and *Brucella*-negative reference sera (Shchelkovo Biocombinet, Moscow, Russia) were used in this study. In addition, monospecific antisera against *B. abortus* and *B. melitensis* (Animal and Plant Health Agency, Weybridge, UK), *Yersinia enterocolitica* O9 (Bio-Rad, California, USA), *Salmonella* spp. (Invitrogen, Carlsbad, CA, USA), and *E. coli* (Ecolab, Moscow, Russia) were employed.

*Brucella*-positive sera were obtained by subcutaneous immunization of rabbits with inactivated *B. abortus* 19 and *B. melitensis* Rev1 cells, as previously described [17]. Blood samples were collected from the marginal ear vein under sedation on day 0 (*Brucella*-negative serum). Hyperimmune serum was collected on day 56 and stored at −20°C until further use.

### Blood serum samples and biological material from animals

A total of 1,440 blood serum samples from productive animals were included in this study. Among them, 92 cattle and 96 sheep serum samples were obtained from brucellosis-affected farms/flocks. In addition, 127 cattle and 46 sheep serum samples were seropositive for brucellosis based on conventional serological tests. A total of 500 cattle and 547 sheep serum samples were classified as *Brucella*-negative. These serum samples (n = 1,408) were provided by the Republican Veterinary Laboratory (RVL) in Kazakhstan and stored at −20°C until analysis. Samples of the liver, spleen, and parotid, retropharyngeal, and submandibular lymph nodes were collected from 32 cattle before sanitary slaughter for culture isolation and polymerase chain reaction (PCR) analysis, and blood serum samples from the same animals were obtained for serological testing.

Herds were classified as brucellosis-affected if at least one animal tested positive in confirmatory serological assays (complement fixation test [CFT] and/or tube agglutination test [TAT]) upon repeated testing, in accordance with national veterinary regulations. Herds were considered brucellosis-free if all animals tested negative in Rose Bengal plate test (RBPT), CFT, and TAT over time and no evidence of infection was detected. Animals were classified as *Brucella*-positive based on consistent positive results in confirmatory serological tests (CFT and/or TAT) upon repeated examination and as *Brucella*-negative if they tested negative in all serological assays (RBPT, CFT, and TAT). Both classifications were further supported by results obtained using a commercial ELISA kit (IDvet, Montpellier, France).

### Preparation of the brucellosis erythrocyte antigen for the IHA

This study introduces a modified tannin-sensitization protocol optimized for recombinant proteins, which differs from traditional LPS-based methods by incorporating rivanol and defined protein concentrations to enhance epitope presentation on erythrocytes [18].

Tannin solution was prepared by dissolving 20 mg of tannin (Thermo Scientific, Kandel, Germany) in 100 mL of distilled water, followed by incubation in a water bath at 37°C for 20 min. A 2.5% suspension of formalinized erythrocytes, previously washed three times with PBS (pH 7.2) by centrifugation at 309 × *g* for 10 min, was mixed with an equal volume of tannin solution. The tannin-treated erythrocytes were subsequently washed three times with PBS under the same centrifugation conditions. The supernatant was discarded, and the pellet was resuspended in PBS (pH 6.4) to obtain a 2.5% erythrocyte suspension. Formalin was then added to a final concentration of 1% for preservation. The formalinized erythrocytes were washed three times with PBS-T by centrifugation at 309 × *g* for 10 min. The pellet was resuspended in PBS and incubated in a water bath at 45°C with intermittent mixing. Upon reaching the target temperature, a series of recombinant protein solutions at concentrations of 50, 100, 200, 400, 800, and 1600 μg/mL in PBS-T were added, followed by the addition of 1% rivanol (PharmAgro, Yaroslavl, Russia) to a final concentration of 0.01%. Sensitization was carried out at 45°C for 2 h with gentle mixing to prevent erythrocyte sedimentation.

Following sensitization, the cells were washed three times with physiological saline to remove excess protein, then centrifuged at 309 × *g* for 10–15 min. After the final centrifugation, the erythrocyte pellet was resuspended in physiological saline to obtain 5%, 10%, and 15% erythrocyte suspensions.

The stability of the erythrocyte diagnostic antigen was assessed by IHA using reference positive and negative sera after 6 and 12 months of storage at 4°C. The antigen remained stable and functionally activerelative, with no loss of agglutination intensity compared to baseline measurements throughout the 12-month observation period, showing reactivity with positive sera up to a titer of 1:3200 and an agglutination score of ≥2 crosses.

### Determination of the optimal concentration of the recombinant antigen and erythrocyte suspension

For this purpose, rabbit anti-*Brucella* serum and rabbit negative serum were diluted 1:20 and heat-inactivated in a water bath at 60–62°C for 30 min. In a 96-well polystyrene U-bottom immunoassay plate (Ningbo Greetmed Medical Instruments Co., Ningbo, China), 50 µL of each serum was dispensed into the wells, followed by the addition of 5 µL of an erythrocyte suspension (5%, 10%, or 15%) sensitized with different doses of recombinant protein (50–1600 µg/mL). The well contents were thoroughly mixed and incubated at 37°C under static conditions. A preliminary observation of the reaction was performed after 2 h; however, final recording and interpretation of the results were carried out only after 18–24 h of incubation, based on the pattern of erythrocyte sedimentation at the bottom of the wells, according to the following scoring system:

(++++) – Strong agglutination characterized by a clearly formed, homogeneous umbrella-like erythrocyte sediment covering the bottom of the well;

(+++) – Moderate agglutination with a smaller umbrella-shaped sediment and a distinct central ring of non-agglutinated erythrocytes;

(++) – Partial agglutination presenting as a uniform ring of settled non-agglutinated cells with a granular peripheral rim;

(+) – Weakly positive reaction observed as a thin brownish-red ring of erythrocyte sediment;

(−) – Negative reaction in which erythrocytes formed a compact button at the bottom of the well with no evidence of agglutination.

A clearly defined positive reaction (3 or 4 crosses) was considered indicative of the optimal concentrations of recombinant protein and erythrocyte suspension.

### Evaluation of the reactivity of the working suspension of erythrocytes sensitized with *Brucella* spp. Antigen (ED)

Two-fold serial dilutions of rabbit anti-*Brucella* serum raised against *B. abortus* and/or *B. melitensis*, as well as rabbit negative serum, were prepared in PBS to a final volume of 100 µL per well in a 96-well polystyrene U-bottom immunoassay plate, starting from a 1:2 dilution in 8 wells. Subsequently, 10 µL of the ED suspension was added to each well. Incubation of the plates and recording of the IHA results were performed in accordance with the procedure described above. Serum samples exhibiting erythrocyte agglutination with an intensity of 2 crosses or higher were considered positive for *Brucella*-specific antibodies.

### Protocol for serological testing of animal sera by IHA using ED based on recombinant antigens

Ninety microliters of PBS (pH 7.4) were dispensed into each well of a 96-well plate, followed by the addition of 10 μL of the test serum. The contents of the wells were thoroughly mixed to ensure homogeneous distribution of the components. Subsequently, 10 μL of the ED was added to each well, and the plates were gently shaken to mix the reagents. Incubation of the plates and recording of the IHA results were performed in accordance with the procedure described above. Serum samples exhibiting erythrocyte agglutination with an intensity of 2 crosses or higher were considered positive for *Brucella*-specific antibodies.

### Serological testing of animal sera by RBPT

Blood serum samples were examined for brucellosis using the RBPT (RPE “Antigen”, Almaty, Kazakhstan), in accordance with Interstate Standard GOST 34105-2017 [19].

### Animal serum analysis by i-ELISA

Recombinant antigens were coated onto 96-well polystyrene microplates (ThermoFisher Scientific) at a concentration of 1.0 μg/mL in CBB (pH 9.6) and incubated overnight at 4°C. Bovine and/or ovine serum samples were added to the wells at a fixed dilution of 1:100 in PBS-T, then incubated at 37°C for 1 h. Horseradish peroxidase-conjugated rabbit anti-bovine IgG and/or donkey anti-sheep IgG (Sigma-Aldrich, St. Louis, MO, USA) were used as secondary antibodies for the detection of bovine and ovine sera, respectively. The cut-off value for i-ELISA was calculated based on negative cattle and sheep sera and defined as the mean OD at 492 nm (OD_492_) plus 3 standard deviations (mean + 3 SD). All samples were analyzed in triplicate.

### Evaluation of cross-reactivity and specificity of *Brucella* proteins

Cross-reactivity of *Brucella* recombinant proteins was assessed by i-ELISA using standard homologous and heterologous antisera described above, as well as *Brucella*-negative reference serum. Antibody titers were defined as the highest serum dilution at which the OD value was at least two-fold higher than that of the negative reference serum tested at a dilution of 1:100. The specificity of the proteins in the IHA was evaluated using a panel of sera collected from animals originating from brucellosis-free herds/flocks and confirmed negative by reference serological assays.

### Diagnostic test evaluation

The number of true-positive (TP) and false-negative (FN) cases by IHA in testing *Brucella*-positive sera and the number of true-negative (TN) and false-positive (FP) cases by IHA in testing *Brucella*-negative sera were counted. From these data, sensitivity (Se), specificity (Sp), and accuracy (Ac) were calculated using the following equations (All expressed as percentages):

Se = TP/(TP + FN);

Sp = TN/(TN + FP);

Ac = (TP + TN)/(TP + TN + FP + FN), (all are expressed as percentages).

### Bacteriological and molecular genetic diagnostic methods

Bacteriological examination of samples, including blood serum samples and tissue specimens (liver, spleen, and parotid, retropharyngeal, and submandibular lymph nodes), collected from cattle seropositive for brucellosis during sanitary slaughter, was performed in accordance with Interstate Standard GOST 33675-2015 [20]. Molecular genetic diagnosis was performed using a real-time PCR assay (Vet-Factor, Moscow, Russia). Suspensions prepared from biological tissue samples were used as primary test material, followed by analysis of aliquots obtained from meat–peptone broth after 10 days of incubation post-inoculation.

### Statistical analysis

Comparisons of mean OD values were performed using Student’s t-test. Statistical significance was defined as p < 0.05. Correlation analysis was conducted using Pearson’s correlation coefficient (r), and the strength of correlations was interpreted according to the Chaddock scale as follows: r = 0.75–1.00, very high positive correlation; r = 0.50–0.74, high positive correlation; r = 0.25–0.49, moderate positive correlation; and r = 0.00–0.24, weak positive correlation. Diagnostic performance parameters (sensitivity, specificity, and accuracy) were calculated as described above, and corresponding 95% confidence intervals (CI) were estimated using the exact (Clopper–Pearson) binomial method.

## RESULTS

### Characterization and antigenicity of recombinant *Brucella* proteins

**Figure 1:**
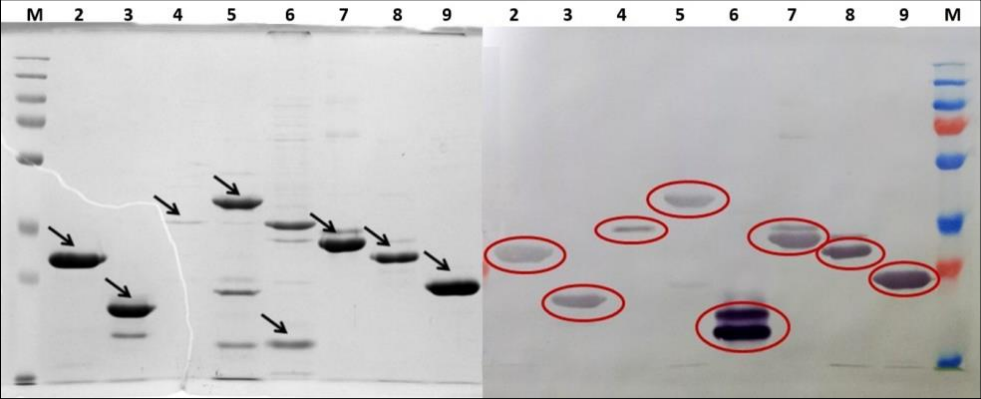
Electropherogram (left) and immunoblot (right) analysis of recombinant *Brucella* spp. M: Protein molecular weight markers; Lane 2: Bp26; Lane 3: Cu-ZnSOD; Lane 4: Omp25; Lane 5: Omp31; Lane 6: Omp19; Lane 7: Omp25+31; Lane 8: Omp19+31; Lane 9: Omp19+25.

Electropherogram and immunoblot of *Brucella* proteins purified by metal chelate chromatography (Ni^2+^) are shown in Figure 1.

Analysis using gel documentation systems (GelDoc™ Go, Bio-Rad, California, USA) and the Photo-Capt software, version 12.4 (Vilber Lourmat, France), demonstrated that the target proteins Omp19, Omp25, Omp31, Omp19+Omp25, Omp19+Omp31, Omp25+Omp31, Cu-ZnSOD, and Bp26 had molecular weights of 18.6, 41.8, 46.8, 23.0, 27.4, 30.4, 20.9, and 27.2 kDa, respectively, as determined by SDS-PAGE. Immunoblotting analysis further confirmed the expression of the recombinant proteins in the producer cells. The increased molecular weights of Omp25 and Omp31 are attributable to the presence of thioredoxin in the pET32 expression vector, which was included to enhance protein solubility.

The antigenicity of the prepared proteins was evaluated by i-ELISA using rabbit sera from animals immunized with inactivated *B. abortus* 19 and *B. melitensis* Rev1 cells (Table 1).

**Table 1:** Antigenicity of recombinant *Brucella* proteins in i-ELISA.

Rabbit anti-serum against	Single Omps			Chimeric Omps			Periplasmic proteins	
Immunogen	19	25	31	19+25	19+31	25+31	Cu-ZnSOD	Bp26
*Brucella abortus* 19	1:400	1:3200	1:200	1:800	1:1600	1:3200	1:1600	1:400
*Brucella melitensis* Rev1	1:400	1:1600	1:400	1:400	1:1600	1:1600	1:800	1:800

Rabbit antisera raised against whole cells of *B. abortus* 19 and *B. melitensis* Rev1 exhibited reactivity with their homologous immunogens at dilutions of up to 1:12,800, confirming the suitability of these antisera for subsequent evaluation. As shown in Table 1, the recombinant proteins differed in their antigenic reactivity: Omp19+31, Omp25+31, and Omp25 were detected at serum dilutions of 1:1,600–1:3,200, whereas the other proteins reacted only at lower dilutions of 1:400–1:800.

### Preparation of erythrocyte diagnostic reagent (ED) for IHA

To determine the optimal conditions for the preparation of ED based on a recombinant antigen and to ensure maximal sensitivity of the IHA, formalinized erythrocytes were sensitized with Omp19 at concentrations ranging from 50 to 1,600 µg/mL using 5%, 10%, and 15% erythrocyte suspensions. The optimal antigen dose was defined as the lowest concentration that produced a clear and reproducible agglutination pattern of sensitized erythrocytes in the presence of rabbit anti-*Brucella* serum (1:20), corresponding to reaction scores of 3 or 4 crosses, while maintaining the absence of agglutination (≤1 cross) with negative rabbit serum. Under these criteria, an Omp19 concentration of 100 µg/mL was identified as optimal, ensuring stable and uniform sensitization of erythrocytes regardless of the suspension concentration used. Among the tested erythrocyte concentrations, the 5% suspension proved to be the most suitable for the IHA, producing a uniform and well-defined agglutinate that was readily distinguishable from a compact erythrocyte button, thereby facilitating reliable interpretation of the results. Higher erythrocyte concentrations resulted in either weak agglutination or excessive sedimentation, without forming a distinct agglutination pattern. Accordingly, a 5% erythrocyte suspension sensitized with the protein at a concentration of 100 µg/mL was selected as the optimal formulation for ED preparation in the IHA protocol.

**Figure 2:**
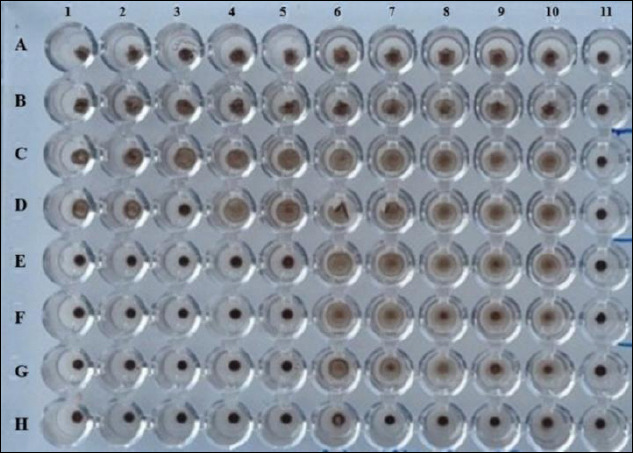
Reactivity of recombinant protein-sensitized ED with rabbit anti-*Brucella* sera. Rows 1 and 6: ED/Cu-ZnSOD; Rows 2 and 7: ED/Bp26; Rows 3 and 8: ED/Omp19; Rows 4 and 9: ED/Omp25; Rows 5 and 10: ED/Omp31. Two-fold serial dilutions of rabbit anti-*B. abortus* and anti-*B. melitensis* sera were prepared and dispensed into the wells of Rows 1–5 and 6–10, respectively, starting at a dilution of 1:2 and proceeding sequentially through eight wells. Row 11 contained two-fold serial dilutions of rabbit negative serum.

The results of the evaluation of ED reactivity based on individual proteins are presented in Figure 2. The results of the IHA demonstrated that ED, consisting of erythrocytes sensitized with the used proteins, exhibited varying reactivity toward *Brucella*-specific antibodies. Specifically, anti-*B. abortus* serum induced agglutination of sensitized erythrocytes at titers of 1:8–1:16, whereas anti-*B. melitensis* serum interacted with the ED at higher dilutions: 1:128 for Cu-ZnSOD, Bp26, and Omp19; 1:64 for Omp31; and 1:32 for Omp25. In contrast, the use of negative control serum did not result in agglutinate formation; instead, the erythrocytes sedimented as a compact “button,” thereby confirming the specificity of the assay.

### Recombinant protein-based IHA in brucellosis-affected herds

The IHA, based on ED adsorbed with Omps or periplasmic proteins, was used to examine blood sera provided by the RVL, Republic of Kazakhstan. The serum samples were obtained from cattle (n = 92) and sheep (n = 96) originating from brucellosis-affected farms and flocks and were supplied without accompanying results of conventional serological testing. These sera were analyzed by IHA, and the results were compared with those obtained by i-ELISA using recombinant *Brucella* proteins and RBPT (Table 2).

**Table-2:** Serological test results and correlation analysis in cattle sera (n = 92). A. Number of seropositive animals

Antigens	Bp26	CuZn-SOD	Omp19	Omp25	Omp31
i-ELISA	42 (45.7%)	29 (31.5%)	70 (76.1%)	57 (62.0%)	70 (76.1%)
IHA	38 (41.3%)	25 (27.2%)	20 (21.7%)	17 (18.5%)	35 (38.0%)

**Table:** B. Correlation coefficients (r) between IHA variants based on different *Brucella* proteins

IHA variants	IHA/Bp26	IHA/CuZn-SOD	IHA/Omp19	IHA/Omp25	IHA/Omp31
IHA/Bp26	1.000	0.552	0.575	0.454	0.798
IHA/CuZn-SOD	0.552	1.000	0.606	0.664	0.602
IHA/Omp19	0.575	0.606	1.000	**0.768**	0.673
IHA/Omp25	0.454	0.664	**0.768**	1.000	0.492
IHA/Omp31	**0.798**	0.602	0.673	0.492	1.000

Bold values indicate the highest seropositivity and the strongest correlations.

The data presented in Table 2 indicate that the detection rate of anti-*Brucella* antibodies by i-ELISA depended on the type of recombinant protein used as the antigen. In this assay, Omp19 and Omp31 exhibited the highest antigenicity, allowing the detection of specific antibodies in 76.1% of cattle. Animal seropositivity, as determined by the recombinant antigen-based IHA, ranged from 18.5% to 41.3%. A moderate positive correlation between i-ELISA and IHA was observed only with Omp31 as the antigen (r = 0.297). Notably, the RBPT yielded positive results in only 13 animals (14.1%) of the examined population. Within this herd, the results of the conventional serological test showed a moderate positive correlation with those of IHA/Omp25 (r = 0.289) and weak positive correlations with IHA/Cu-ZnSOD (r = 0.231) and IHA/Omp19 (r = 0.164).

A high degree of concordance was observed among the IHA results obtained using erythrocytes sensitized with different proteins. Very high positive correlations were detected between IHA/Omp25 and IHA/Omp19 (r = 0.768) and between IHA/Omp31 and IHA/Bp26 (r = 0.798). High positive correlations were also observed between the following IHA variants: Omp19–Omp31 (r = 0.673), Cu-ZnSOD–Omp25 (r = 0.664), Omp19–Cu-ZnSOD (r = 0.606), Omp31–Cu-ZnSOD (r = 0.602), Bp26–Omp19 (r = 0.575), and Bp26–Cu-ZnSOD (r = 0.552). At the same time, the IHA/Bp26–IHA/Omp25 and IHA/Omp31–IHA/Omp25 combinations showed a moderate positive association, with correlation coefficients of r = 0.454 and r = 0.492, respectively.

When serum samples from sheep in affected flocks were examined, a positive reaction to brucellosis by RBPT was detected in only 3 animals (3.1%); meanwhile, flock seropositivity in the IHA with respect to Omp31 (13.5%), Omp19 (14.6%), Omp25 (15.6%), and Bp26 (27.1%) was higher (Table 3).

**Table 3:** Serological test results and correlation analysis in sheep sera (n = 96). A. Number of seropositive animals.

Antigens	Bp26	CuZn-SOD	Omp19	Omp25	Omp31
i-ELISA	16 (16.7%)	20 (20.8%)	27 (28.1%)	17 (17.7%)	**41 (42.7%)**
IHA	26 (27.1%)	3 (3.1%)	14 (14.6%)	15 (15.6%)	13 (13.5%)

**Table:** B. Correlation coefficients (r) between IHA variants based on different *Brucella* proteins.

IHA variants	IHA/Bp26	IHA/CuZn-SOD	IHA/Omp19	IHA/Omp25	IHA/Omp31
IHA/Bp26	1.000	0.203	0.111	0.147	0.253
IHA/CuZn-SOD	0.203	1.000	0.622	**0.767**	0.673
IHA/Omp19	0.111	0.622	1.000	**0.744**	0.621
IHA/Omp25	0.147	**0.767**	**0.744**	1.000	0.716
IHA/Omp31	0.253	0.673	0.621	**0.716**	1.000

Bold values indicate the highest seropositivity and the strongest correlations.

In i-ELISA, as in cattle, the highest proportion of seropositive sheep was observed when Omp19 (28.1%) and Omp31 (42.7%) were used as antigens. The analysis of correlation relationships showed that IHA/Omp25 was strongly positively correlated with IHA/CuZn-SOD (r = 0.767), IHA/Omp19 (r = 0.744), and IHA/Omp31 (r = 0.716). A high positive correlation was noted between the IHA variants in cases where CuZn-SOD and Omp31 (r = 0.673), CuZn-SOD and Omp19 (r = 0.622), and Omp19 and Omp31 (r = 0.621) were used as antigens.

### Performance of recombinant antigen-based IHA and i-ELISA in seropositive animals

The sensitivity of the IHA was evaluated using sera from cattle (n = 95) and sheep (n = 46) with laboratory-confirmed brucellosis, diagnosed by the RVL according to a two-stage protocol in accordance with the Veterinary and Sanitary Rules of the Republic of Kazakhstan. Initial screening was performed by RBPT, with confirmation by CFT (at a serum dilution of 1:5). Samples yielding positive or doubtful results underwent second-stage testing, including the standard tube agglutination test (SAT) and CFT (1:5 and 1:10) at species-specific serum dilutions. These sera were provided without available data on the outcomes of individual serological assays.

**Figure 3:**
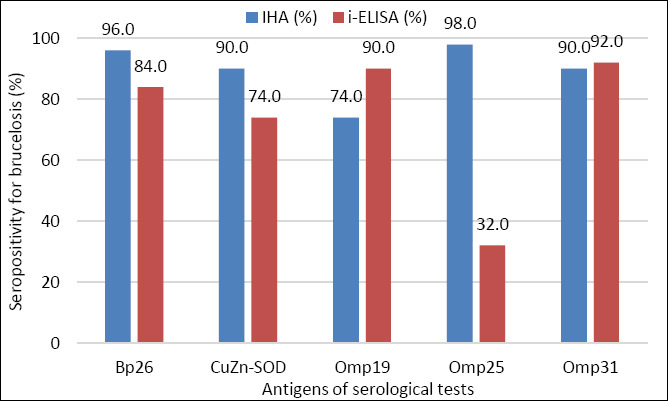
Sensitivity of protein-based IHA and i-ELISA in brucellosis-positive cattle.

Figure 3 presents the sensitivity of the IHA and i-ELISA using different *Brucella* proteins in cattle serum samples.

In our study, RBPT yielded positive reactions in 62 (65.3%) cattle of the examined herd. The sensitivity of the IHA was >90% when Bp26 and Omp25 were used as antigens. A relatively high sensitivity of i-ELISA was observed when Omp19 and Omp31 were used as antigens (≥90%). A strong positive correlation was observed between the IHA variants based on the periplasmic proteins Bp26 or CuZn-SOD and Omp31 (r = 0.691 and r = 0.710, respectively). In addition, the results obtained with the IHA variants based on the two periplasmic proteins showed a strong correlation (r = 0.634). A moderate correlation was detected between the antigenicity of Omp25 and Omp31 (r = 0.286), as well as between Omp25 and Bp26 (r = 0.385).

**Figure 4:**
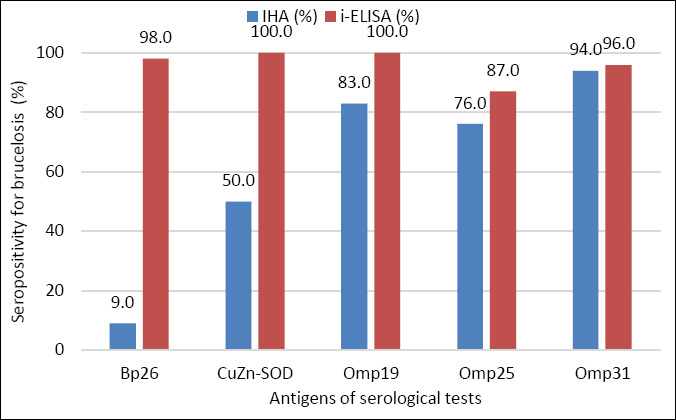
Sensitivity of protein-based IHA and i-ELISA in brucellosis-positive sheep.

Figure 4 shows the sensitivity of IHA and i-ELISA based on *Brucella* proteins in the study of blood sera of brucellosis-positive sheep (n = 46).

The RBPT, performed before the examination of sera by IHA and i-ELISA, yielded positive reactions in 32 (70%) animals of the examined herd. In the IHA, the highest antigenicity was observed for Omp31 (43 animals, 94%) and Omp19 (38 animals, 83%). These same antigens, together with periplasmic proteins, conferred high sensitivity to the i-ELISA (96–100%). Moderate (r = 0.386–0.423) and strong (r = 0.665) positive correlations were observed among the IHA variants. Very high and high correlations between i-ELISA formats were detected when Omp19 and Omp31 (r = 0.777) and Bp26 and Omp31 (r = 0.667) were used as antigens, respectively. Thus, in sera from cattle and small ruminants with laboratory-confirmed brucellosis, diagnosed according to national Veterinary and Sanitary Rules, Omp19 and Omp31 conferred high sensitivity to IHA and i-ELISA. In contrast, periplasmic proteins, while providing high sensitivity of IHA when tested with cattle sera, exhibited low antigenicity in sheep sera. Strong correlations among IHA variants indicate good agreement between assays using different recombinant antigens.

### Cross-reactivity and specificity of *Brucella* proteins

**Figure 5:**
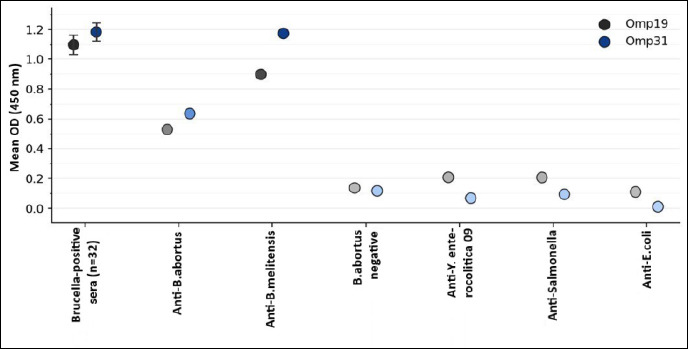
Evaluation of cross-reactivity of *Brucella* proteins using antisera against related microorganisms.

For further analysis, Omp19 and Omp31 were selected as the most suitable antigens for the IHA. The cross-reactivity of the selected proteins was evaluated by i-ELISA using homologous and heterologous commercial sera and 32 serum samples collected from serologically positive cattle prior to sanitary slaughter (Figure 5).

As shown in Figure 5, both Omps demonstrated specificity for *Brucella* spp. and may therefore be used as suitable antigens in serological assays. For example, when Omp19 was used, the OD values in wells containing standard monospecific anti-*B. abortus* and anti-*B. melitensis* sera exceeded those obtained with antisera against *Y. enterocolitica* O:9, *Salmonella* spp., and *E. coli* by 2.6–4.8-fold and 4.3–8.2-fold, respectively. When Omp31 was employed in the i-ELISA, these differences were more pronounced, with fold increases in OD reaching 6.8–63.5 and 12.6–117.3, respectively. The mean OD values of wells sensitized with Omp19 or Omp31 after incubation with *Brucella*-positive sera (n = 32) were comparable (1.097 ± 0.064 and 1.183 ± 0.061, p > 0.05) and exceeded those of control wells by 8.1- or 10.2-fold, respectively.

Specificity of IHA was evaluated using a panel of sera obtained from cattle (n = 500) and sheep (n = 547) originating from brucellosis-free herds/flocks that tested negative for brucellosis by reference serological tests (Table 4).

**Table 4:** Specificity of single protein-based IHA in cattle and sheep. Cattle (n = 500)

Omp	Negative (-)	Positive (+)	(++)	(+++)	(++++)	Specificity (%)
Omp19	211 (42.2%)	258 (51.6%)	27 (5.4%)	4 (0.8%)	0 (0%)	93.8
Omp31	223 (44.6%)	178 (35.6%)	88 (17.6%)	10 (2.0%)	1 (0.2%)	80.2

**Table:** Sheep (n = 547)

Omp	Negative (-)	Positive (+)	(++)	(+++)	(++++)	Specificity (%)
Omp19	114 (20.8%)	334 (61.1%)	82 (15.0%)	15 (2.7%)	2 (0.4%)	81.9
Omp31	146 (26.7%)	280 (51.2%)	102 (18.6%)	19 (3.5%)	0 (0%)	77.9

ED IHA = Erythrocyte Diagnostic Indirect Hemagglutination Assay. Specificity is calculated based on negative results. Bold values indicate the highest specificity.

As shown in Table 4, the IHA using Omp19-based ED demonstrated higher specificity than Omp31 in both cattle (93.8% vs. 80.2%) and sheep sera (81.9% vs. 77.9%) for the detection of anti-*Brucella* antibodies. In contrast, i-ELISA performed on the same sera using Omp19 or Omp31 yielded markedly higher specificity for *Brucella* spp. than the IHA, reaching 98.2% and 96.2% in cattle and 98.6% and 95.7% in sheep, respectively (data not shown).

### Antigenicity and diagnostic performance of combined *Brucella* proteins

Despite the high correlation between IHA variants based on *Brucella* Omps, discrepancies were observed in certain cases, whereby the results obtained with IHA/Omp31 were not confirmed by those of IHA/Omp19 and/or IHA/Omp25, and vice versa. Therefore, to enhance the diagnostic informativeness of the IHA, composite *Brucella* antigens comprising immunogenic regions of paired proteins—namely Omp19+31, Omp19+25, and Omp25+31—were evaluated.

All tested combinations of recombinant proteins exhibited comparable antigenic reactivity toward *Brucella*-positive cattle sera (n = 95), as evidenced by similar mean OD values (0.396 ± 0.102, 0.383 ± 0.093, and 0.304 ± 0.090, respectively) and the absence of statistically significant differences between groups. Nevertheless, the ratio of the OD of test sera to that of the control serum varied with the combined proteins, with Omp19+31 showing significantly higher values than the other formulations, indicating more distinct discrimination between test and control sera (p < 0.05). This advantage justified the selection of the Omp19+31 combination for ED sensitization. In our previous study, this chimeric protein also proved to be a more promising antigen in the i-ELISA [21].

**Figure 6:**
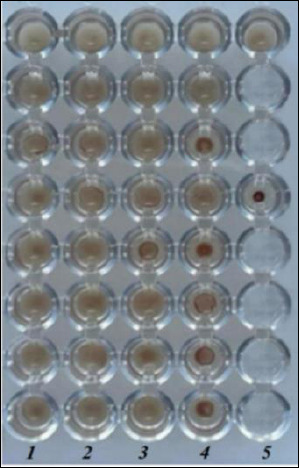
Results of the IHA using combined antigen in brucellosis-positive cattle. Serum samples (n = 32) were arranged sequentially from Well 1 of Row 1 to Well 8 of Row 4. Row 5 contained control sera, including positive serum (Well 1) and negative serum (Well 4). Agglutination was assessed using a semi-quantitative scoring system. Strong positive reactions (++++) were characterized by the formation of a homogeneous umbrella-like erythrocyte layer covering the bottom of the well, whereas negative reactions (−) formed a compact erythrocyte button. Intermediate reactions (+++, ++, and +) reflected progressively weaker agglutination patterns characterized by partial umbrella formation, ring-shaped sedimentation, or a thin peripheral erythrocyte ring.

The results of testing 32 cattle classified as brucellosis-positive based on a combination of diagnostic methods for hemagglutinins against Omp19+31 are shown in Figure 6.

*B. abortus* was successfully isolated from the lymph nodes of only 3 animals (9.4%), and the bacteriological findings were confirmed by PCR. RBPT and CFT detected *Brucella*-specific antibodies in all culture-positive animals, except for 1 animal (No. 8), which tested seronegative by both assays. The commercial ELISA kit likewise failed to detect specific antibodies in this animal and additionally yielded a negative result for another culture-positive animal.

The IHA based on the combined antigen, detected *Brucella*-specific antibodies in all tested samples, with strong positive reactions (4 crosses) observed in 26 animals (81.3%), including No. 8; moderate reactions (3 crosses) in 3 animals (9.4%); and weakly positive reactions (2 crosses) in 3 animals. The Omp19+31-based IHA showed an apparent sensitivity of 100.0% (95% CI: 89.1–100.0), compared with 96.9% for RBPT/CFT (95% CI: 83.8–99.9) and 93.8% for ELISA (95% CI: 79.2–99.2). The sensitivity of the combined antigen-based IHA (100%) exceeded that of assays employing Omp31 (96.8%) or Omp19 (80.6%) individually (data not shown). Thus, the Omp19+31 chimeric antigen-based IHA uniquely detected antibodies in all 32 seropositive cattle (including culture-positive cases missed by commercial ELISA), with predominantly strong (4+) agglutination reactions, a performance not previously reported for recombinant protein-sensitized IHA.

Table 5 summarizes the performance characteristics of IHA and i-ELISA assays using individual and combined *Brucella* proteins, demonstrating their ability to discriminate between seropositive (n = 95) and seronegative (n = 500) cattle.

**Table 5:** Diagnostic performance of i-ELISA and IHA using *Brucella* antigens.

Antigen / Test	Sensitivity, % (95% CI)	Specificity, % (95% CI)	Accuracy, % (95% CI)
Omp19 i-ELISA	90.0 (82.8–95.6)	98.2 (96.6–99.2)	97.0 (95.5–98.1)
Omp31 i-ELISA	90.0 (82.8–95.6)	96.2 (94.1–97.7)	95.3 (93.3–96.9)
Omp19+31 i-ELISA	**100.0 (96.2–100.0)**	92.6 (89.9–94.7)	94.5 (92.3–96.1)
Omp19 IHA	74.0 (63.6–82.2)	93.8 (91.3–95.7)	90.6 (88.0–92.8)
Omp31 IHA	90.0 (84.1–96.3)	80.2 (76.4–83.6)	82.0 (78.8–85.0)
Omp19+31 IHA	92.6 (85.5–96.4)	**100.0 (99.2–100.0)**	**98.8 (97.5–99.5)**

The Omp19+31 combination achieved perfect specificity (100%) in IHA while maintaining high sensitivity, demonstrating complementary advantages over the same antigens in i-ELISA format.

Sensitivity was calculated using 95 seropositive cattle sera, specificity using 500 seronegative cattle sera, and accuracy using all 595 cattle sera.

CI were calculated using the exact (Clopper–Pearson) binomial method.

Best overall performance: Omp19+31 IHA (highest accuracy + perfect specificity)

Highest sensitivity: Omp19+31 i-ELISA (100%). Bold values indicate the highest sensitivity.

As shown in Table 5, Omp19+31 exhibited higher sensitivity in i-ELISA than the individual proteins, albeit at the expense of reduced specificity. In contrast, in the IHA, this antigen proved more advantageous, providing the highest test specificity. A sensitivity-specificity trade-off was also evident within the IHA: Omp19 primarily contributed to assay specificity, whereas Omp31 enhanced sensitivity. The rational design of a chimeric protein incorporating immunodominant regions of both Omp19 and Omp31 enabled the development of an optimized antigen for IHA, achieving 100% specificity and an overall diagnostic accuracy of 98,8%.

To further assess the potential field performance of the assays, positive predictive values (PPV) and negative predictive values (NPV) were estimated from the observed sensitivity and specificity at assumed brucellosis prevalence levels of 1% and 5%, reflecting low-prevalence conditions typical in Kazakhstan. The results are presented in Table 6.

**Table 6:** Estimated positive and negative predictive values of i-ELISA and IHA at assumed brucellosis prevalence levels.

Antigen/Test	Se, %	Sp, %	PPV at 1% prevalence, %	NPV at 1% prevalence, %	PPV at 5% prevalence, %	NPV at 5% prevalence, %
Omp19 i-ELISA	90.0	98.2	33.6	99.9	72.5	99.5
Omp31 i-ELISA	90.0	96.2	19.3	99.9	55.5	99.5
Omp19+31 i-ELISA	**100.0**	92.6	12.1	**100.0**	41.6	**100.0**
Omp19 IHA	74.0	93.8	10.8	99.7	38.6	98.6
Omp31 IHA	92.0	80.2	4.5	99.9	19.7	99.5
Omp19+31 IHA	92.6	**100.0**	**100.0**	99.9	**100.0**	99.6

Se = Sensitivity, Sp = Specificity. PPV = Positive predictive value, NPV = Negative predictive value. Values are calculated at disease prevalence levels of 1% and 5%. Omp19+31 IHA shows excellent performance with perfect PPV at both prevalence levels due to 100% specificity.

The results indicate that predictive values are strongly influenced by disease prevalence. The IHA, based on the Omp19+31 antigen, maintained a PPV of 100% across both scenarios because of its high specificity, while the NPV remained above 99%. In contrast, assays with lower specificity showed markedly reduced PPV, particularly at low-prevalence levels. These results demonstrate that, under low-prevalence conditions, assay specificity is a key determinant of PPV, whereas NPV remains consistently high across tests. The Omp19+31-based IHA, because of its maximal specificity, is therefore particularly suitable for confirmatory use in endemic settings.

## DISCUSSION

### Diagnostic significance of recombinant protein-based IHA

The present study demonstrates, to our knowledge, the first successful adaptation of recombinant *Brucella* outer membrane proteins, particularly the chimeric Omp19+Omp31 construct, as sensitizing antigens in an IHA for the serological diagnosis of animal brucellosis. In contrast to conventional LPS-based IHA formats, which are prone to cross-reactivity and have encountered practical limitations in certain settings, the protein-based approach developed here offers improved specificity while preserving the inherent simplicity, cost-effectiveness, and visual interpretability of IHA. Collectively, these findings represent a methodological advance by integrating the field applicability of traditional IHA with the molecular specificity of recombinant antigens previously validated predominantly in ELISA-based platforms.

Key contributions of this study include: (1) a systematic comparison of single versus chimeric Omps in IHA, indicating that Omp19+31 is among the most diagnostically informative formats; (2) demonstration of format-dependent performance differences between IHA and i-ELISA when using identical antigens; (3) evaluation of diagnostic performance under field-relevant endemic conditions using a large sample set from both cattle and sheep; and (4) evidence that the assay can detect antibodies in cases where culture and commercial ELISA yielded negative or equivocal results. These findings extend current understanding and help address an important diagnostic gap in brucellosis-endemic countries with limited laboratory infrastructure.

### Previous applications and limitations of conventional IHA

The IHA is a non-reference serological method with considerable potential for diagnosing animal brucellosis. Its diagnostic utility has been extensively investigated since the 1970s [22, 23], including evaluations of various *Brucella*-sensitizing antigens, erythrocyte sources, and ED preparation methods [24]. The assay is particularly effective at early stages of infection, when conventional tests may yield equivocal or negative results [22,25], and is based on the agglutination of antigen-coated erythrocytes with anti-*Brucella* antibodies in serum [26–28] or milk [29, 30]. Owing to its simplicity, reproducibility, and adequate analytical sensitivity, IHA has been implemented in veterinary practice, including industrial-scale production and routine use in the Russian Federation since 2007, and remains regulated by the Interstate Standard GOST 34105–2017.

However, the commercial IHA kit based on *Brucella* LPS (Vetmedservice, Makhachkala, Republic of Dagestan, Russia) is limited by low specificity, and the manufacturer has discontinued its production. Cross-reactivity of anti-*B. abortus* 99 hemagglutinins with *Y. enterocolitica* O:9 in IHA was described during the analysis of sera from patients with chronic brucellosis [31]. FP IHA reactions have also been described in tularemia (post-infection or vaccination) and other infectious diseases when erythrocytes sensitized with *B. abortus* 19 LPS were used, reflecting shared surface antigens among Gram-negative bacteria and highlighting a major limitation of LPS-based IHA antigens [32]. Similar FP reactions have been documented in animals with bovine tuberculosis [33], melioidosis [34], and cystic echinococcosis [35]. In addition, substantial inter-laboratory variability in IHA results has been reported, even when identical sera were tested [36].

### Antigenic characteristics and assay optimization

Standardization and widespread implementation of IHA for brucellosis diagnosis require highly specific antigens and reproducible, scalable production platforms. Recombinant *Brucella* outer membrane and periplasmic proteins have demonstrated superior specificity [37–39], and their diagnostic potential has been summarized in our recent review [40]. The present findings confirm that, when based on recombinant antigens, IHA can serve as a simple, specific, and standardized serological test. Its particular relevance for endemic regions with limited diagnostic resources has been emphasized previously [41].

SDS-PAGE and immunoblotting confirmed correct protein expression, while i-ELISA revealed antigen-dependent differences in reactivity, with combined recombinant proteins (Omp19+31 and Omp25+31) showing enhanced antibody binding compared with several individual antigens.

Optimization of erythrocyte sensitization showed that antigen concentration and erythrocyte density are key determinants of IHA performance. Recombinant proteins were evaluated in IHA for the first time in this study, building upon their prior successful application in i-ELISA. Recent reviews on IHAs have highlighted the need for standardized approaches using well-defined recombinant antigens to improve diagnostic specificity and reproducibility [41]. Sensitization at 100 µg/mL with a 5% erythrocyte suspension provided clear agglutination without nonspecific reactions.

The IHA revealed antigen-dependent differences in ED reactivity toward *Brucella*-specific antibodies. ED based on Cu-ZnSOD, Bp26, and Omp19 showed higher reactivity with anti-*B. melitensis* serum than Omp31 and Omp25, whereas all antigens reacted weakly with anti-*B. abortus* serum. The consistently higher titers of anti-*B. melitensis* serum, despite identical immunization conditions, likely reflect epitope dominance driven by antigen processing and presentation mechanisms [42]. This suggests that the immunodominant epitopes of the recombinant proteins more closely match those targeted during immunization with *B. melitensis* cells.

It should be noted that the antigenicity of the same protein varied depending on both the type of antiserum and the serological assay employed. For example, in i-ELISA, Omp19 was detected by both anti-*B. abortus* and anti-*B. melitensis* sera at a titer of 1:400, whereas in the IHA the titers differed markedly, reaching 1:8 and 1:128, respectively. In i-ELISA, the titers of both antisera against Omp31 did not exceed 1:200, whereas those against Omp25 ranged from 1:1,600 to 1:3,200. In contrast, in the IHA no substantial differences in titers were observed for either protein.

The results demonstrate that the antigenicity of recombinant proteins varies depending on both the type of antiserum and the serological assay format. The differences observed between i-ELISA and IHA are most likely attributable to methodological features of these assays and the mode of antigen presentation. In i-ELISA, antigen adsorption to a solid phase may favor exposure of linear epitopes, resulting in comparable anti-B titers*. abortus* and anti-*B. melitensis* sera against Omp19 and higher reactivity of Omp25. In contrast, antigen immobilization on erythrocytes in IHA preserves spatial epitope organization and requires multivalent antigen-antibody interactions, which likely explains the marked differences in Omp19 titers between antisera, while no substantial differences were observed for Omp31 and Omp25.

### Diagnostic performance under field conditions

Studies conducted in brucellosis-affected herds and flocks demonstrated a higher diagnostic performance of the recombinant protein-based IHA compared with RBPT, which identified seropositive animals in only 14.1% of cattle and 3.1% of sheep, respectively, whereas IHA detected anti-*Brucella* antibodies in 18.5–41.3% of cattle and 13.5–27.1% of sheep, depending on the antigen used.

Here, we acknowledge that seropositive animals may have been incompletely identified by RBPT due to limited sensitivity under certain field conditions, particularly in animals with chronic infection or low antibody titers [43]. Therefore, the observed differences between RBPT and the recombinant protein-based IHA may be attributed to several factors. On the one hand, RBPT, although widely used as a screening test, may exhibit reduced sensitivity depending on the stage of infection and the dynamics of the antibody response. On the other hand, the IHA developed in this study, based on recombinant protein antigens, may detect a broader spectrum of anti-*Brucella* antibodies, including those not efficiently recognized by LPS-based assays.

Consequently, the higher detection rates observed with IHA may reflect either limitations in RBPT sensitivity or the enhanced ability of the recombinant protein-based assay to identify protein-specific antibody responses. However, in the absence of a uniform reference standard for all animals, these interpretations should be considered with caution. As in most brucellosis studies, the reference classification in the present work was based on composite serological criteria rather than a single definitive gold standard, a distinction that should be taken into account when interpreting diagnostic performance estimates and comparing results across studies.

In accordance with WOAH guidelines, the validation of diagnostic assays should be guided by the intended use (fitness for purpose) and supported by appropriate evaluation of analytical and diagnostic performance using well-characterized sample panels [44]. Recent systematic reviews and meta-analyses suggest that, although serological assays such as ELISA remain central to brucellosis diagnosis, their performance may vary across epidemiological settings, and the use of combined testing approaches can improve overall diagnostic reliability [45,46]. In this context, the proposed IHA may be considered a complementary assay within existing diagnostic frameworks.

A high degree of concordance was observed among IHA variants sensitized to different recombinant proteins, as evidenced by strong-to-very-strong positive correlations across multiple assay formats. The consistency of these inter-IHA correlations indicates stable, reproducible antibody detection despite antigenic variability, underscoring the assay’s robustness. Collectively, these findings support the use of recombinant protein-based IHA as a complementary serological tool to enhance brucellosis surveillance in affected herds, particularly under field conditions where the sensitivity of conventional tests is limited.

### Comparative performance of Omps and periplasmic proteins

The present findings indicate that Omps, particularly Omp19 and Omp31, exhibit consistently high diagnostic performance in IHA for laboratory-confirmed brucellosis in both cattle and small ruminants. In contrast to periplasmic proteins, these Omps exhibited balanced and reliable reactivity across host species, whereas the periplasmic antigens showed acceptable sensitivity in cattle sera but markedly reduced antigenicity in sheep. This reduced antigenicity in sheep is likely attributable to species-specific features of the humoral immune response during *B. melitensis* infection, in which antibodies are predominantly directed against surface-exposed antigens. Conversely, cattle infected mainly with *B. abortus* appear to develop a more balanced antibody response to both surface and periplasmic antigens, resulting in satisfactory reactivity in IHA.

This interspecies consistency highlights the suitability of the Omps as universal antigens for IHA-based serological screening. Furthermore, the strong correlations observed among IHA formats using different recombinant antigens support the assay’s robustness and indicate that these Omps contribute to stable, reproducible test performance.

It should be noted that the sensitivity of the IHA was assessed using sera from animals classified as *Brucella*-positive by the RVL based on RBPT, SAT, and CFT, in which S-LPS of whole cells constitutes the primary antigen. Therefore, differences in antigenic targets between LPS-based reference assays and the protein-based IHA may influence the apparent sensitivity estimates of the latter.

### Performance of the chimeric Omp19+31 antigen

The opposite specificity patterns observed for Omp19 and Omp31 in i-ELISA and IHA reflect format-dependent differences in antigen presentation. In i-ELISA, immobilization on polystyrene surfaces favors the exposure of *Brucella*-specific epitopes and reduces nonspecific binding, resulting in higher Omp31 specificity. In contrast, antigen adsorption on erythrocytes in IHA may alter epitope accessibility and promote background reactivity, under which Omp19 exhibits lower nonspecific interactions and higher specificity. Thus, the differential performance of Omp19 and Omp31 is determined by assay format rather than intrinsic antigen quality.

Despite the high concordance among IHA formats based on individual *Brucella* Omps, no single protein alone captured the full spectrum of specific antibodies. This prompted us to evaluate the diagnostic performance of composite Omps as antigens in the IHA. Previously, we showed that these chimeric proteins exhibit stronger antigenicity than the individual Omps in i-ELISA [14] and that Omp19+31 outperformed the other combined formats [21]. In this study, this antigen also provided higher diagnostic informativeness for the IHA than the other composite proteins.

The assay was evaluated using clinical samples collected from seropositive cattle during sanitary slaughter. *Brucella*-specific antibodies were detected by RBPT and CFT in 96.9% of animals and by a commercial ELISA kit in 93.8%, whereas bacteriological confirmation was obtained in only 9.4% of cases. This discrepancy is consistent with the well-recognized limitations of culture-based diagnosis in livestock [44]; therefore, culture negativity does not rule out infection and supports the need for complementary serological approaches.

Against this background, the IHA/Omp19+31 demonstrated higher analytical sensitivity, detecting antibodies in all tested sera and predominantly yielding strong agglutination reactions; its sensitivity exceeded that of the corresponding single-antigen IHA formats. The combined recombinant antigen yielded complementary performance in i-ELISA and IHA. i-ELISA achieved 100% sensitivity with 92.6% specificity, whereas IHA showed 92.6% sensitivity with 100% specificity, consistent with format-dependent differences in antigen presentation and background reactivity.

### Predictive values, limitations, and future perspectives

Predictive values further illustrate the assay’s expected performance under field conditions and highlight the practical importance of assay specificity in low-prevalence settings. At assumed brucellosis prevalence levels of 1% and 5%, assays with suboptimal specificity demonstrated a marked decline in positive predictive value, indicating a high proportion of FP results. In contrast, the Omp19+31-based IHA maintained a PPV of 100% across both prevalence scenarios due to the absence of FP reactions, underscoring its suitability for confirmatory testing in endemic regions. At the same time, the consistently high NPV (>99%) observed across all assays indicates reliable exclusion of infection in test-negative animals. Collectively, these findings emphasize that in epidemiological contexts with low disease prevalence, diagnostic specificity is a critical determinant of the practical utility of serological assays.

A limitation of the present study is that the potential to differentiate infected from vaccinated animals (DIVA capability) was not evaluated, as sera from vaccinated but uninfected animals were not available. This aspect is particularly important in endemic regions where vaccination is practiced and will require dedicated investigation in future studies. In addition, the study did not include a formal assessment of intra- and inter-assay reproducibility, including inter-operator and inter-laboratory variability.

While building on our previous work with these chimeric proteins in ELISA, this study represents one of the first applications in IHA, opening avenues for standardized, scalable protein-based ED reagents.

## CONCLUSION

The present study demonstrated the successful development and evaluation of a recombinant protein-based IHA for the serological diagnosis of animal brucellosis using recombinant *Brucella* Omps and periplasmic proteins as sensitizing antigens. Among the evaluated antigens, the chimeric Omp19+31 construct exhibited the most favorable diagnostic performance, combining high sensitivity with maximal specificity in the IHA format. The developed assay effectively detected anti-*Brucella* antibodies in cattle and sheep sera from endemic regions of Kazakhstan and demonstrated higher diagnostic informativeness than the conventional RBPT under field conditions. Notably, the Omp19+31-based IHA detected antibodies in all tested seropositive cattle, including cases not detected by commercial ELISA, demonstrating its potential as a complementary confirmatory serological method.

The findings further showed that the diagnostic performance of recombinant proteins was strongly influenced by the assay format and the mode of antigen presentation. Omps, particularly Omp19 and Omp31, provided stable and reproducible reactivity across different host species, whereas periplasmic proteins exhibited species-dependent antigenicity. The incorporation of immunodominant regions from multiple proteins into a chimeric construct improved the assay’s diagnostic characteristics and enhanced discrimination between positive and negative sera.

From a practical perspective, the developed recombinant protein-based IHA offers several important advantages for veterinary diagnostics, including operational simplicity, visual interpretability, low equipment requirements, and suitability for large-scale screening in laboratories with limited infrastructure. These characteristics make the assay particularly relevant for brucellosis-endemic countries where access to advanced diagnostic platforms may be restricted. The high specificity of the Omp19+31-based IHA also suggests its usefulness as a confirmatory assay under low-prevalence conditions, where minimizing FP results is critically important for preventing unnecessary culling of animals.

A major strength of the present study is the comprehensive evaluation of both single and chimeric recombinant antigens using a large collection of cattle and sheep sera obtained under field-relevant endemic conditions. In addition, the study systematically compared the performance of recombinant antigens in both IHA and i-ELISA formats, thereby providing new insights into format-dependent antigen behavior and diagnostic applicability.

However, several limitations should be acknowledged. The study did not evaluate the assay’s ability to differentiate infected from vaccinated animals (DIVA capability) because sera from vaccinated but uninfected animals were unavailable. Furthermore, a formal assessment of intra- and inter-laboratory reproducibility was not performed. The reference classification of animals was based primarily on composite serological criteria rather than a single definitive gold standard, a consideration when interpreting diagnostic performance estimates.

Future studies should focus on large-scale, multicenter validation of the assay across different epidemiological settings, assessment of reproducibility and long-term stability, and evaluation of DIVA applicability in vaccinated populations. Additional investigation of alternative chimeric recombinant proteins and optimization of antigen combinations may further improve assay sensitivity and broaden its diagnostic utility.

Overall, the present findings indicate that recombinant Omp-based IHA, particularly the Omp19+31 format, represents a promising, specific, and field-applicable serological tool for the diagnosis and surveillance of animal brucellosis. The assay may serve as a valuable complementary approach within existing diagnostic frameworks and contribute to more reliable brucellosis control programs in endemic regions.

## DATA AVAILABILITY

The data generated during the study are included in the manuscript.

## AUTHORS’ CONTRIBUTIONS

AB: Conceptualization, interpretation of the results, manuscript editing. AS: Data curation, validation, and writing – original draft. AK and GK: Testing of sera by IHA and i-ELISA and statistical analysis. AZ and MB: Sample collection, culture isolation, and PCR analysis. MM: Methodology and study design. BI: Recombinant protein production and purification. All authors contributed to the study design, critically reviewed the manuscript, and approved the final version of the manuscript.
